# Case of the month from the University of Sheffield, UK: Expediting definitive treatment in patients with invasive bladder cancer: an MRI‐guided pathway

**DOI:** 10.1111/bju.15730

**Published:** 2022-05-28

**Authors:** Samantha Conroy, Rachel Hubbard, Aidan P. Noon, Syed A. Hussain, Jon Griffin, Steven Kennish, James W. F. Catto

**Affiliations:** ^1^ Academic Urology Unit, Department of Oncology and Metabolism University of Sheffield Sheffield UK; ^2^ Department of Urology Sheffield Teaching Hospitals NHS Foundation Trust Sheffield UK; ^3^ Department of Radiology Sheffield Teaching Hospitals NHS Foundation Trust Sheffield UK; ^4^ Academic Oncology Unit, Department of Oncology and Metabolism University of Sheffield Sheffield UK; ^5^ Department of Histopathology Sheffield Teaching Hospitals NHS Foundation Trust Sheffield UK

**Keywords:** bladder cancer, clinical pathways, magnetic resonance imaging, transurethral resection of bladder tumour, VI‐RADS

## Case Presentation

A 77‐year‐old gentleman was referred with a raised PSA (8.9 μg/L) and two episodes of painless visible haematuria. He was fit, with an ECOG performance status of 0, had never smoked, and had no occupational risk factors for bladder cancer (BC). At the time of referral, his baseline haemoglobin was 154 g/L, and he had a creatinine of 92 μmol/L and eGFR of 69 mL/min/1.73 m^2^. A contrast computed tomography (CT) scan, arranged by his GP, identified a 5 cm solid‐looking tumour on the right lateral wall of the bladder that was not causing ureteric obstruction. He was promptly referred to our haematuria service, attending 6 days after his CT scan. Flexible cystoscopy was performed with informed consent, revealing a large, solid looking bladder tumour.

**Fig. 1:**
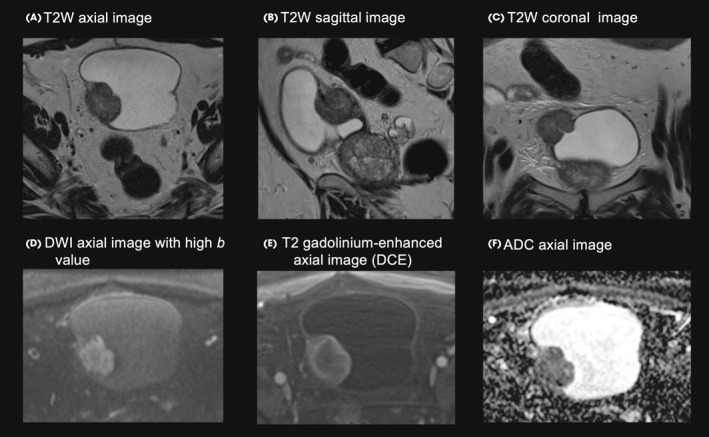
T2W, DWI, DCE and ADC mpMRI image sequences of a large VI‐RADS 4 bladder tumour. ADC, apparent diffusion coefficient; DCE, dynamic contrast‐enhanced imaging; DWI, diffusion‐weighted imaging; T2W, T2‐weighted imaging.

**Fig. 2:**
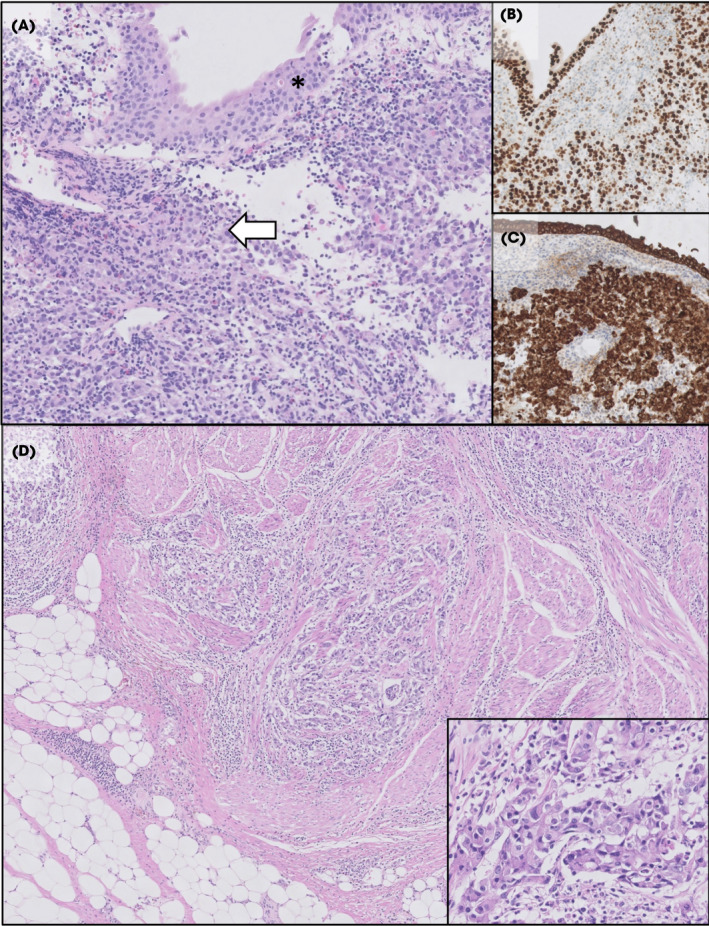
Histopathology of biopsy and cystectomy specimens. (**A**) Haematoxyllin and eosin (H&E) stained biopsy showing malignant cells in the lamina propria (white arrow) underneath the urothelium (asterisk). (**B** and **C**) Tumour cells with nuclear GATA3 staining and membranous AE1/AE3 staining respectively. (**D**) H & E section from the cystectomy showing tumour invading muscle with adjacent peri‐vesical adipose tissue. Inset image shows tumour cell detail. [Colour figure can be viewed at wileyonlinelibrary.com]

After being counselled about the diagnosis and discussing the next steps in the clinical pathway, he consented to take part in a clinical trial exploring the feasibility of image‐directed redesign of the BC treatment pathway: the BladderPath study [1] . A biopsy was taken at the time of flexible cystoscopy and this confirmed a high‐grade urothelial cell carcinoma (plasmacytoid variant) and at least lamina propria invasion (G3pT1+). He was randomised to the multiparametric magnetic resonance imaging (mpMRI) assessment arm of the study [1]. On mpMRI imaging, the tumour appeared to be muscle invasive with a VI‐RADS score of 4 [2]. There were no size‐significant metastatic lymph nodes in the pelvis and no apparent extra‐vesical extension (Figs 1, 2).

## Management Considerations

Despite recent advances in the field [3], survival outcomes from invasive BC have not improved for 30 years [4]. This may reflect the biology of the disease, healthcare behaviour of the at risk population [5] or that treatment improvements are needed. With regards to the latter, current pathways for BC patients can be slow and fail to discriminate between non‐muscle invasive (NMIBC) and muscle‐invasive cancers (MIBC) [6, 7]. For patients with MIBC, guidelines recommend that RC should not be delayed for longer than 12 weeks (84 days), as longer delays have significant impact on survival [8]. Data from the Urology *Getting it right first time* (GIRFT) national report that in 2013–2016 patients with MIBC were waiting an average of 144 days from referral to definitive therapy within the NHS [9]. A BAUS Radical Cystectomy audit, from 2017–2019, suggested the delay was even longer (with 48% of patients waiting >180 days from diagnosis to RC [10]). Considering the increased service demand pressures that COVID has created, current delays to definitive treatment for patients with MIBC may be even longer. Hence, we feel there is a need to rethink the MIBC pathway.

TURBT has been at the heart of BC diagnosis and treatment for the best part of a century. Advocates detail the procedure can treat symptoms through debulking, may remove all NMIBC, is rapid, allows histological staging, and should be safe. However, the expanse and accessibility of non‐invasive imaging, coupled with concerns over the safety of TURBT [11, 12], question its role within MIBC. TURBT in MIBC is primarily a staging tool in patients eligible for radical treatment. Both GIRFT [9] and NICE (NICE guideline NG2, 2019)[13] have encouraged a rethink of NHS targets, such that TURBT is no longer taken as a treatment milestone.

In this case, a patient with suspected MIBC had a risk‐adapted, image‐triaged pathway that combined flexible cystoscopic biopsy with mpMRI assessment. mpMRI and standardised nomenclature (such as Prostate Imaging‐Reporting and Data System) have improved prostate cancer management, although its role in BC is currently under evaluation. VI‐RADS (Vesical Imaging‐Reporting and Data System) was designed to help protocolise mpMRI imaging, and assist radiologists in the systematic evaluation and standardised reporting of bladder mpMRI [2]. VI‐RADS uses a combination of T2‐weighted imaging (T2W), diffusion‐weighted imaging (DWI), dynamic contrast‐enhanced imaging (DCE) and apparent diffusion coefficient (ADC), to evaluate the risk of muscle invasion using a five‐point scale (VI‐RADS 1 – muscle invasion highly unlikely, to VI‐RADS 5 – muscle invasion highly likely). High quality bladder MRI imaging, as with all imaging of the bladder, is largely dependent on good bladder distension. Allowing for this, the high resolution T2 weighted imaging (ideally in 3 planes) is the dominant sequence for assessing interruption of the low T2 signal muscular layer by tumour. Individual tumour characteristics determine the respective added values of DCE and DWI sequences.

## Treatment Course

**Fig. 3:**
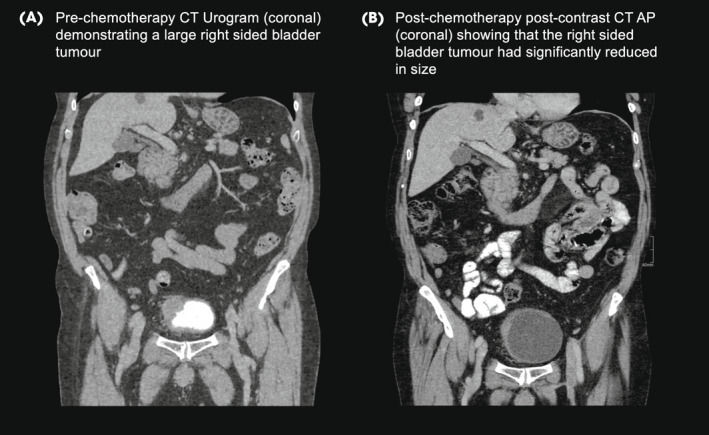
Pre and post‐neoadjuvant chemotherapy computed tomography (CT) appearances of the right sided muscle invasive bladder tumour.

As per the BladderPath trial protocol, the patient was offered immediate radical treatment without TURBT. He agreed with this approach and started four cycles of neoadjuvant gemcitabine, cisplatin and guadecitabine – a novel DNA methyltransferase inhibitor within the SPIRE trial [14] – 43 days after his diagnostic flexible cystoscopy. His post‐chemotherapy CT scan revealed the tumour had reduced in size from 5.0 to 2.2 cm, with no evidence of disease spread, but was still suspicious for muscle invasion. After treatment for incidental bilateral pulmonary emboli, he underwent RC and lymphadenectomy without further complication. Final pathology revealed a pT3a, non‐metastatic (0 from 9 nodes) urothelial carcinoma of the bladder with clear margins, and a Gleason 3 + 3 = 6 pT2aN0R0 adenocarcinoma of the prostate. His most recent surveillance CT scan, at 33 months after referral, has shown no evidence of disease recurrence or metastasis, and his PSA was undetectable (Fig. 3).

## Conclusion

Within many healthcare systems, BC diagnostic and treatment pathways require re‐evaluation to ensure that they can discriminate between NMIBC and MIBC patients in a timely manner. The expansion of non‐invasive imaging – including mpMRI of the bladder – provides scope to deliver a non‐invasive, diagnostic pathway that can help to expedite the radical treatment of MIBC patients. The VI‐RADS system provides a framework to facilitate standardised reporting and urological understanding of risk. Although further validation is needed, the success and widespread implementation of mpMRI in prostate cancer pathways suggests that this imaging modality is feasible and robust. In this case, mpMRI assessment suggested that MIBC was likely and in conjunction with high‐grade histology and flexible cystoscopy appearance, provided a decision‐making adjunct to rapidly triage this gentleman to radical therapy (NAC). He is currently disease‐free almost 3 years after referral.

## Disclosure of Interests

JWFC and SAH are co‐investigators in the Phase I SPIRE Trial of DNA Methyltransferase Inhibitor Guadecitabine Combined with Cisplatin and Gemcitabine for Solid Malignancies funded by ECMC Combination Alliance which includes funding from Cancer Research UK (C9317/A19903) and investigator‐initiated research support from Astex Pharmaceuticals. JWFC and SK are co‐investigators on the BladderPath study funded by the National Institute for Health Research Health Technology Assessment Programme (project number 14/08/60). JWFC is funded by a National Institute for Health Research Professorship (2019–24). JG is funded by a Clinical PhD Fellowship from the Pathological Society of Great Britain and Ireland and the Jean Shanks Foundation (JSPS‐CPHD‐2018‐01). SC was previously funded by The Urology Foundation 2020–2021 *via* their Research Scholars Award (award number 200520).

**Abbreviations**
BC: bladder cancer
DCE: dynamic contrastenhanced
DWI: diffusion‐weighted imaging
GIRFT: Getting it Right First Time
MIBC: muscle‐invasive bladder cancer
mpMRI: multiparametric MRI
NMIBC: non‐muscle‐invasive bladder cancer
TURBT: transurethral resection of bladder tumour
VI‐RADS: Vesical Imaging‐Reporting and Data System
